# Advances and Applications of Polygenic Scores for Coronary Artery Disease

**DOI:** 10.1146/annurev-med-042921-112629

**Published:** 2022-10-31

**Authors:** Aniruddh P. Patel, Amit V. Khera

**Affiliations:** 1 Division of Cardiology and Center for Genomic Medicine, Department of Medicine, Massachusetts General Hospital, Boston, Massachusetts, USA; 2 Cardiovascular Disease Initiative, Broad Institute of MIT and Harvard, Cambridge, Massachusetts, USA; 3 Department of Medicine, Harvard Medical School, Boston, Massachusetts, USA; 4 Verve Therapeutics, Cambridge, Massachusetts, USA

**Keywords:** polygenic score, coronary artery disease, genomics, risk prediction, risk factors, prevention

## Abstract

Polygenic scores quantify inherited risk by integrating information from many common sites of DNA variation into a single number. Rapid increases in the scale of genetic association studies and new statistical algorithms have enabled development of polygenic scores that meaningfully measure—as early as birth—risk of coronary artery disease. These newer-generation polygenic scores identify up to 8% of the population with triple the normal risk based on genetic variation alone, and these individuals cannot be identified on the basis of family history or clinical risk factors alone. For those identified with increased genetic risk, evidence supports risk reduction with at least two interventions, adherence to a healthy lifestyle and cholesterol-lowering therapies, that can substantially reduce risk. Alongside considerable enthusiasm for the potential of polygenic risk estimation to enable a new era of preventive clinical medicine is recognition of a need for ongoing research into how best to ensure equitable performance across diverse ancestries, how and in whom to assess the scores in clinical practice, as well as randomized trials to confirm clinical utility.

## INTRODUCTION

Identification of individuals at high risk for coronary artery disease (CAD)—the leading cause of death globally—to enable targeted prevention therapies remains a major public health need (1). The inherited component of risk of CAD was recognized as early as 1951, when close relatives of an afflicted family member were reported to have an approximately two-fold increase in risk (2-5). Subsequent studies of twins suggested that the inherited component of CAD (“heritability”) may be up to 60% (6-8). Early investigation into the genetic basis of CAD involved careful family-based studies, which ultimately identified monogenic forms of the disease related to rare mutations in specific genes (9-11). Here, the prototypical example relates to mutations causal for familial hypercholesterolemia, a condition associated with impaired clearance of low-density lipoprotein (LDL) cholesterol from the circulation. Familial hypercholesterolemia variants are identified in approximately 2% of individuals with premature CAD and 0.4% of the general population (12-14).

Over the past two decades, the focus of study has shifted to the role of common genetic variants in predisposing to CAD. Genetic association studies conducted in more than one million individuals over the past decade have identified over 240 independent variants associated with risk of CAD (15-18). The variants discovered from these large-scale studies are common in the population and confer smaller differences in risk, typically associated with a <5% change in risk per inherited allele. Polygenic scores—which integrate information from many sites of common DNA variation—provide a quantitative metric of inherited risk that integrates information from many such variants into a single number. The concepts of polygenic prediction were first used in plant and animal breeding as early as the 1940s and continue to be used to maximize milk production in the dairy industry (19, 20). These scores have more recently been applied to human traits and diseases.

## FUNDAMENTALS OF SCORE CONSTRUCTION

**Figure 1:**
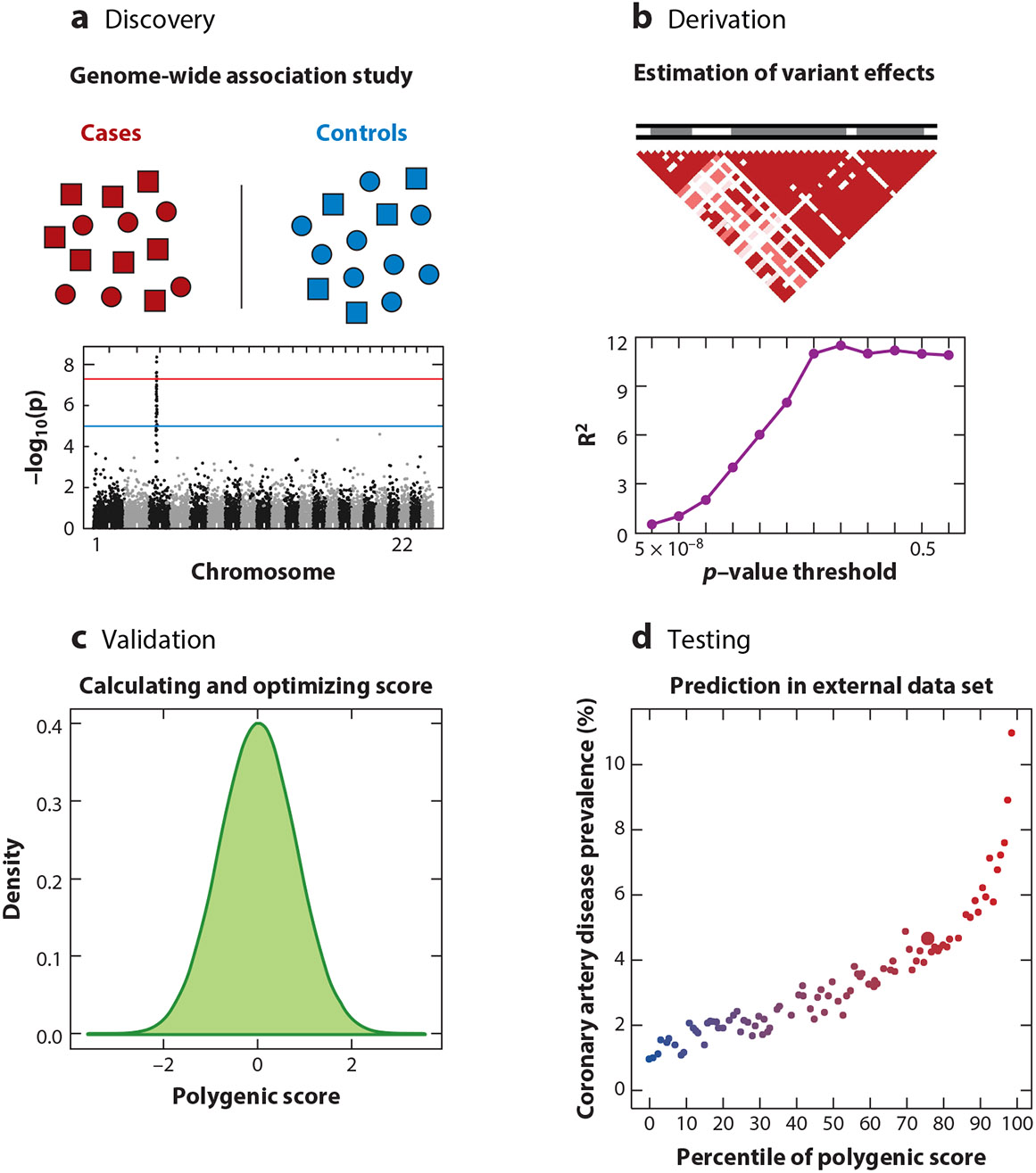
Polygenic score construction. (*a*) Genome-wide association studies generate summary statistics to be used in polygenic score development. (*b*) Linkage disequilibrium patterns and significance of variant association are taken into account when selecting variants for score calculation. (*c*) Summing of effect sizes of each variant across the genome for each individual results in a normal distribution of scores in a population. (*d*) Polygenic scores stratify risk for coronary artery disease, with prevalence of disease in 100 groups binned according to percentile of polygenic score. Figure adapted with permission from Reference 12.

Results from genome-wide association studies (GWAS)—which aim to precisely quantify the relationship between each common DNA variant and disease risk—are the primary input data into the construction of polygenic scores. Common DNA variants are assessed in study participants across millions of sites in their genome. Investigators then compare frequencies of each variant occurring in cases versus controls, thereby generating an output of summary statistics that consists of a directional effect size per allele associated with the disease outcome and a measure of statistical significance (21) (Figure 1a).

Polygenic scores are calculated by summing the effect sizes of risk variants identified in an individual to get a single score, which generally forms a normal distribution when aggregated in the population. Starting with the most significant variants, sequentially higher *p*-value thresholds for inclusion of variants in the score are tested to identify the best-performing overall score (22). Initial efforts focused on only the variants with the strongest statistical associations; however, for polygenic diseases such as CAD, it was recognized that inclusion of variants with less significant associations was of value in improving polygenic score prediction (Figure 1b).

Linkage disequilibrium—the nonrandom association of variants at different locations in the genome—plays a key role in the application of GWAS data in polygenic score construction and needs to be accounted for to minimize noise. Briefly, the process of crossing over of chromosomes occurs during meiosis at recombination sites across the genome. This results in large blocks of genetic sequence patterns that segregate and are inherited together—haplotype blocks. Knowledge of these patterns is used to impute the sequences of DNA at certain sites within the vicinity of directly genotyped sites. Linkage disequilibrium patterns and haplotype blocks vary between ancestries. When constructing scores, it is important to prune out the variants within the same haplotype block as the strongest association to avoid double-counting risk conferred by a set of DNA variants that tend to be inherited together (Figure 1b).

The past decade has seen numerous advancements in the methods used to calculate polygenic scores from the traditional scoring approach and from pruning and thresholding (23). These methods use different statistical techniques to adjust the effect sizes measured from GWAS to reduce polygenic score error and improve performance (24-28). With a growing number of individuals included in GWAS for CAD over the past decade, the predictive power of the scores generated has continued to grow (12, 29-40). From 2010 to 2018, the odds ratio per standard deviation has increased from 1.23 to 1.72 due to a 3.5-fold increase in discovery cases and controls (Figure 1d). Recently developed methods that focus on refining the polygenic score weights based on fine mapping or the functional repercussions of certain variants have also been employed (41-47). Because functional annotations do not differ across ancestral backgrounds, polygenic scores based on this approach may have improved generalizability across ancestries. Other newly developed methods also incorporate GWAS data from multiple ancestries or multiple related traits to improve prediction (48-53).

The validation and testing of polygenic scores must occur in a data set independent from the data sets used in the discovery GWAS from which polygenic score weights are estimated. A subset of the target data is allocated for training, where polygenic score weights are optimized, calculated for a given trait, and benchmarked against other scores (54, 55). The best-performing score is chosen using measures of discrimination to then apply to the testing cohort for targeted risk prediction (Figure 1c,d). The Polygenic Score Catalog has been developed as a database of published scores which allows for cross-application and evaluation of different scores (56).

## RISK PREDICTION

Polygenic scores hold considerable promise for enabling a tailored approach to clinical medicine. The advent of large biobanks that link genetic data with clinical outcomes has proven critical in the development and assessment of polygenic scores (57-62). An important example of such a resource is the UK Biobank, which is a prospective cohort study with extensive genetic, lifestyle, and linked clinical data in >500,000 individuals (63, 64).

**Figure 2:**
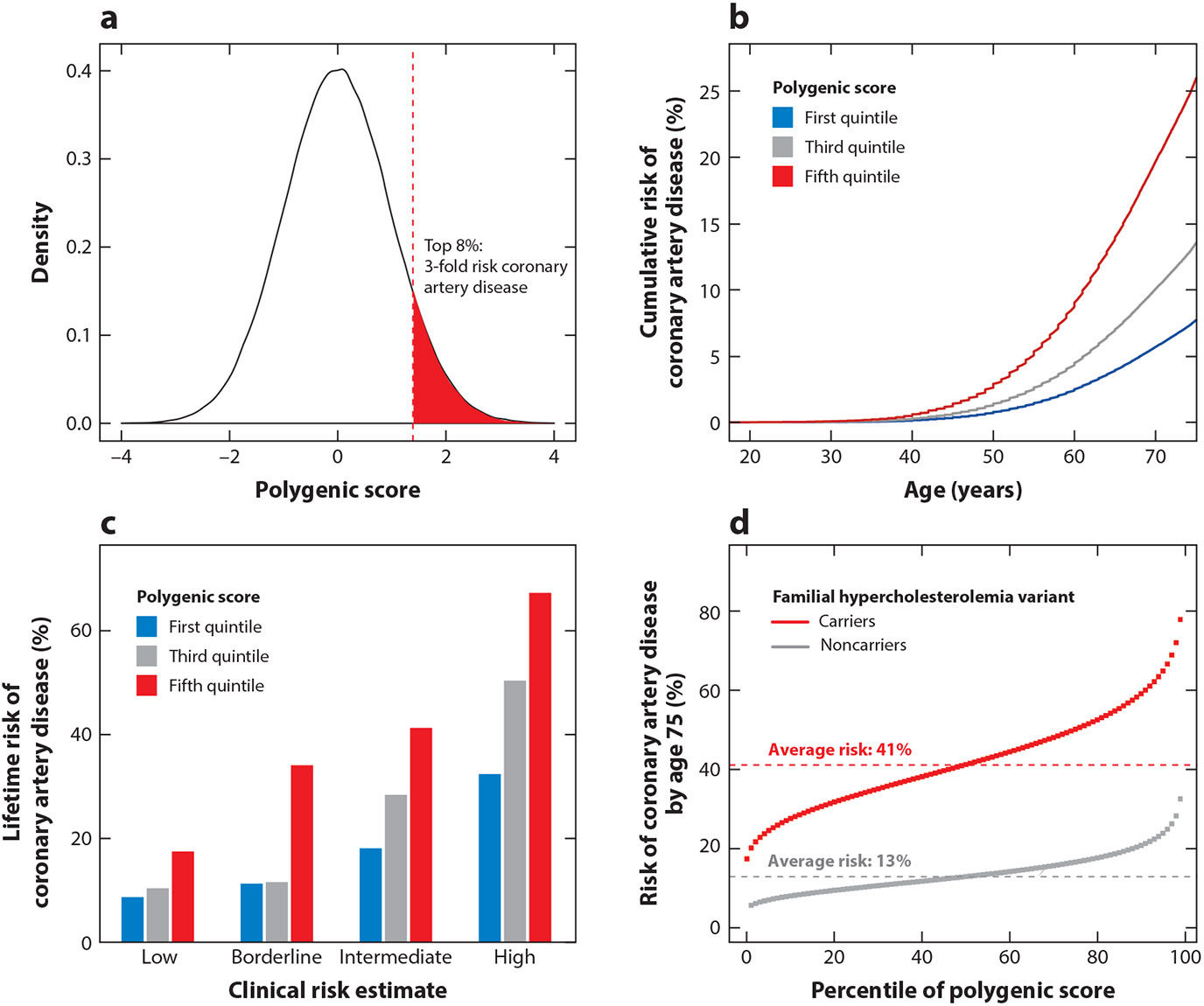
Utility of polygenic scores. (*a*) Distribution of a polygenic score with shading reflecting the proportion of the population with threefold increased risk for prevalent coronary artery disease (CAD) versus the remainder of the population. (*b*) Cumulative risk of CAD by age 75 stratified by quintiles of the polygenic score distribution. (*c*) Cumulative risk of CAD by age 90 stratified by Pooled Cohort Equations risk category. (*d*) Predicted probability of CAD by age 75 in each percentile of the polygenic score distribution stratified by carrier status for a familial hypercholesterolemia variant. Horizontal dashed lines show the probability of disease for people with average polygenic score. Panel (*a*) from Reference 12, (*b*) from Reference 31, (*c*) from Reference 66, (*d*) from Reference 81. All adapted with permission.

Within the UK Biobank, several studies have demonstrated striking gradients in risk of CAD according to polygenic scores. A score constructed using 6.6 million genetic variants and well-powered GWAS summary statistics was able to predict CAD prevalence with an odds ratio of 1.72 per standard deviation in participants of European ancestry (12). This approach identified 8% of the population at threefold increased risk for CAD (Figure 2a). This prevalence is 20-fold higher than the carrier frequency of rare familial hypercholesterolemia variants conferring comparable risk. Other studies have stratified individuals by their cumulative risk for incident disease based on their decile of the score distribution (31) (Figure 2b). The individuals with high genetic risk identified by these methods are “flying under the radar” in current clinical practice, despite evidence that targeted screening or prevention efforts can markedly attenuate polygenic risk. Across three US healthcare systems, individuals in the top quintile of the polygenic score, when compared with the rest of the population, did not meet criteria for increased statin therapy per the American College of Cardiology/American Heart Association recommendations (46.2% versus 46.8%, respectively) or have higher statin prescription rates (25.0% versus 23.8%, respectively) (65).

Polygenic scores capture risk information that is largely independent of traditional risk factors (66). For example, the Pearson correlation coefficients between risk estimated by the Pooled Cohort Equations and polygenic score for CAD ranged from 0.01 to 0.03 across prior studies (66, 67) (Figure 2c). Of the genome-wide significant variants identified through large-scale CAD GWAS, only approximately 10% relate to known traditional risk factors such as lipid levels and blood pressure (68, 69). Although the remaining variants may not have understood mechanisms, they nonetheless offer additional predictive power when incorporated into genetic scoring. Polygenic scores have a higher discriminative capacity for incident CAD in baseline models including age and sex (change in C-statistic 0.045) when compared with 11 traditional risk factors (0.007 to 0.032) (66). Although there is a modest improvement in discrimination when polygenic scores are combined with risk estimates from the Pooled Cohort Equations, the combination of age, sex, and polygenic score alone demonstrates equivalent predictive power to this clinical risk estimator (70). Polygenic scores stratify CAD risk even among individuals with similar predicted risk according to current risk algorithms. Among individuals at borderline risk (5–7.5% 10-year risk estimate by Pooled Cohort Equations), polygenic scores stratified risk further, with lifetime risk ranging from 11.3% in the bottom quintile to 34.1% in the top quintile. Most, but not all, studies have shown improvement in risk prediction resulting from models that incorporate clinical variables and polygenic scores, as compared with clinical variables alone (31, 70-75). A key advantage of a polygenic score is that it can be assessed very early in life, before the onset of traditional risk factors. CAD polygenic scores perform better in younger individuals than in older individuals because at younger ages genetic determinants likely play a larger role in explaining variance with respect to other risk factors (71). An analysis of the polygenic basis of obesity and body weight across the lifespan illustrated the ability to stratify individuals into very different trajectories of cardiometabolic health. Analysis of a prospective cohort study that followed participants from birth to early adulthood noted minimal differences in birth weight across polygenic score deciles; however, this gradient widened through childhood so that by 18 years of age, a 26-pound average difference in weight was noted between the top and bottom deciles (76). Similarly, polygenic scores for CAD assessed in young adulthood stratify individuals along a substantial gradient in risk of future coronary artery calcification and CAD events (77).

Polygenic scores offer an opportunity to refine genetic risk prediction even for those who inherit a familial hypercholesterolemia variant, who have long been recognized in clinical practice to have highly variable expression of disease. A portion of this variation is mediated by a polygenic background (78-80). For carriers of pathogenic variants for hypercholesterolemia, who on average exhibit triple the risk of CAD borne by noncarriers, substantial gradients in CAD risk exist, with the probability of disease by age 75 ranging from 17% to 78% (81). Therefore, familial hypercholesterolemia variant carriers with polygenic scores in the lowest quintile are at a near-population-average CAD risk. Moving forward, state-of-the-art genome interpretation is likely to involve integration of monogenic and polygenic risk into a single report for a complete and accurate picture of common disease risk (82, 83) (Figure 2d).

## ACTIONABILITY OF POLYGENIC SCORES

**Figure 3:**
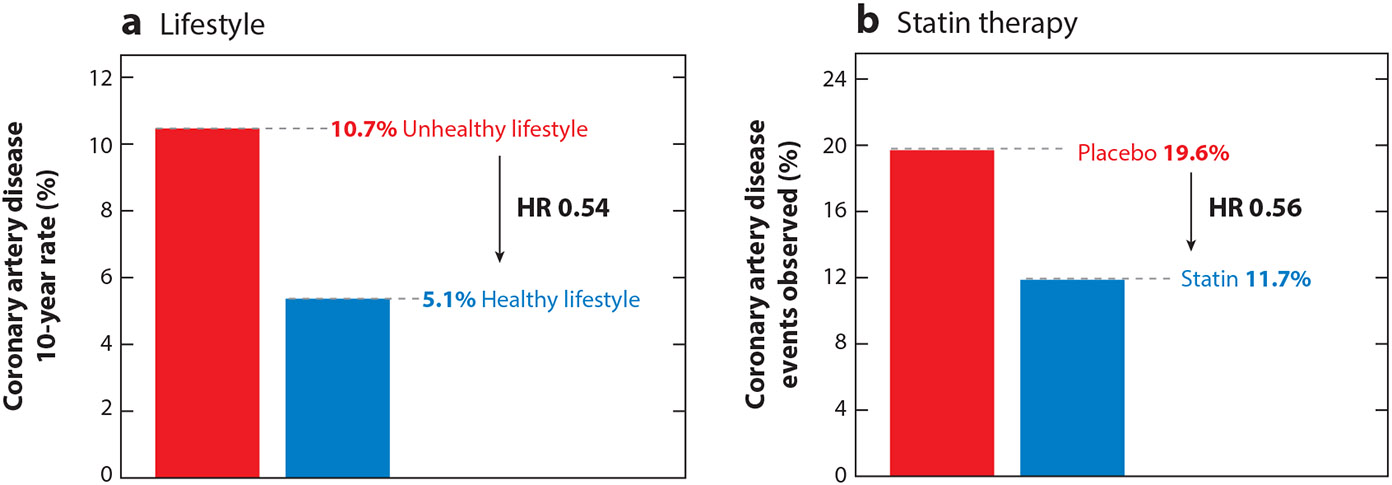
Benefit of lifestyle and therapeutic interventions on genetic risk. Abbreviation: HR, hazard ratio. (*a*) Standardized 10-year cumulative incidence rates for coronary artery disease (CAD) events in individuals with the highest quintile of the polygenic score, stratified according to lifestyle (84). (*b*) Incident CAD event rates in clinical trial participants with the highest quintile of the polygenic score, stratified by statin therapy (89).

Once a high-risk individual is identified based on their polygenic score, several studies have shown they may stand to benefit from preventive interventions. In observational studies, adherence to a healthy lifestyle—specified as no current smoking, no obesity, regular physical activity, and a healthy diet—was associated with an approximately 50% lower relative risk of CAD across the spectrum of polygenic scores compared to those with unfavorable lifestyle characteristics. Individuals with the highest polygenic risk showed the greatest absolute risk reduction (5.6%) from adherence to a healthy lifestyle so as to match the average population-level risk (84, 85) (Figure 3a). Similar to healthy lifestyle, post hoc analysis of trials of cholesterol-lowering therapies also noted increased absolute risk reduction among individuals with high polygenic scores (86-89). In a meta-analysis of primary CAD prevention trials, statin therapy was associated with a relative risk reduction of 46% and absolute risk reduction of 3.6% in the highest genetic risk group, compared to relative risk reduction of 26% and absolute risk reduction of 1.3% across all other groups (89) (Figure 3b).

Early efforts to study the impact of polygenic score disclosure have generally noted mixed results of clinical utility, providing an opportunity for additional investigation. In a prospective study of individuals randomized to receive CAD risk estimates with or without polygenic scores, individuals informed of their polygenic scores had higher rates of statin prescription and lower LDL cholesterol at 6 months of follow-up (90). Another small study did not find change in lipids or behavior after disclosure of a polygenic score result (91). Further study of polygenic score implementation in large randomized controlled trials is needed to guide treatment recommendations based on polygenic scores.

The ability to identify very high-risk individuals using a polygenic score suggests value for clinical trial development. Estimating a polygenic score for potential participants prior to enrollment and selection of individuals within the highest risk categories would enrich for CAD event incidence, reducing the needed sample size and maintaining the same statistical power. This could contribute to substantial savings in clinical trial costs and ultimately in drug costs (92).

Polygenic scores can be used to identify causal pathways using the principles of Mendelian randomization. As genetic variants are randomly assorted during meiosis and are independent of confounders, their association with both a risk factor and a disease is consistent with a causal pathway between the risk factor and the disease. Multiple studies have shown that polygenic scores allow for cross-trait prediction and causal inference (93). For example, a polygenic score optimized for predicting body mass index is also able to detect significant risk for a range of other cardiometabolic diseases including CAD (76). Genetic scores constructed using variants in genes associated with plasma high-density lipoprotein (HDL) cholesterol levels demonstrate no causal association between certain HDL-raising mechanisms and CAD (94). By contrast, similar analyses using variants influencing triglyceride concentrations did suggest a causal relationship with CAD (95).

Polygenic scores for a variety of key conditions can be assessed using a single test with a genotyping array or low-coverage whole-genome sequencing at a technical cost of less than $50. A blood or saliva sample collected early in life would allow for a polygenic susceptibility “report card” including numerous polygenic scores for many diseases. As with CAD, polygenic score methodologies have proven highly generalizable across traits, identifying up to 6.1%, 3.5%, and 1.5% of European individuals at greater than threefold risk for atrial fibrillation, type 2 diabetes, and breast cancer, respectively (12). Polygenic scores may be cost-effective when applied to other diseases, although more work needs to be done to assess this for CAD (96).

## CURRENT LIMITATIONS OF POLYGENIC SCORE GENERALIZABILITY

Alongside considerable enthusiasm for polygenic scores, limitations exist with regard to their generalizability to different populations. A key equity issue has arisen in that although current scores are associated with increased risk across different ancestries, they have diminished predictive power in non-European ancestries (13, 97-99). Approximately 80% of currently available GWAS data are from individuals of European ancestry, and these discovery data sets are not fully informative for generating polygenic scores in individuals of other ancestries due to differences in linkage disequilibrium patterns, allele frequencies, heritability, and genetic architecture (100). To address these challenges, important ongoing efforts aim to gather GWAS data in individuals of non-European ancestry as well as to develop polygenic scoring methods that incorporate multiancestry data or adjust variant weights to leverage shared genetic variation between ancestries (50, 51, 101-107).

## FUTURE DIRECTIONS OF POLYGENIC SCORES

**Figure 4:**
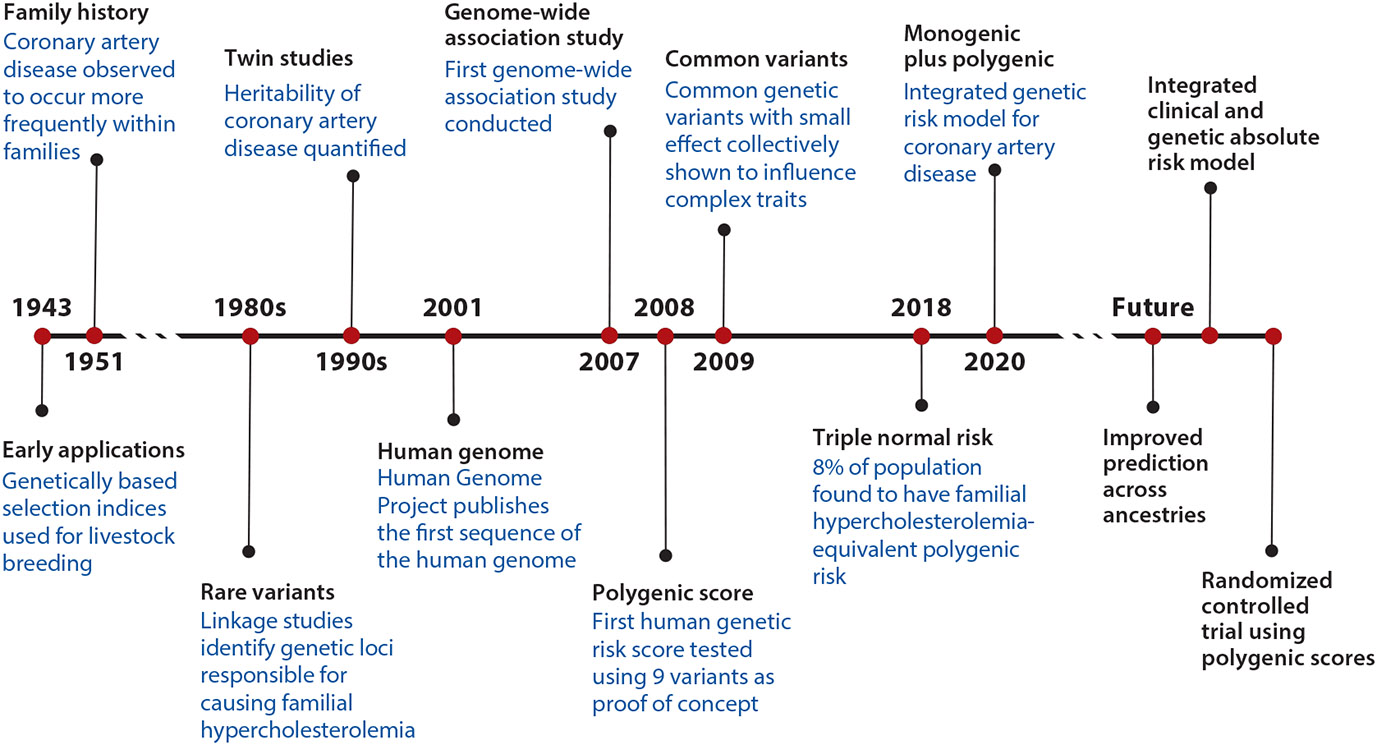
Timeline of advances in the development of polygenic scores.

As polygenic scores continue to improve in portability and accuracy, several key steps remain before they can be incorporated into clinical practice (Figure 4). First, integration of genetic risk with clinical factors into a unified risk prediction model would harness the influence of both primordial and acquired risk factors. Initial efforts have combined information from established clinical risk calculators with polygenic scores to determine an absolute 10-year CAD event rate; however, further work is needed in calibrating integrated risk models to target populations (74, 108, 109). Although polygenic risk is orthogonal and additive to conventional risk factors, characterizing gene-by-risk-factor interaction across subgroups of age and sex remains a challenge, as does grounding prediction in absolute risk estimates. Similarly, unifying reference distributions for ancestry-based comparisons will be key to accurately calculating risk in different populations. Additionally, as more genomic discovery data are acquired, polygenic scores for a given disease will change and need to be updated over time.

**Figure 5:**
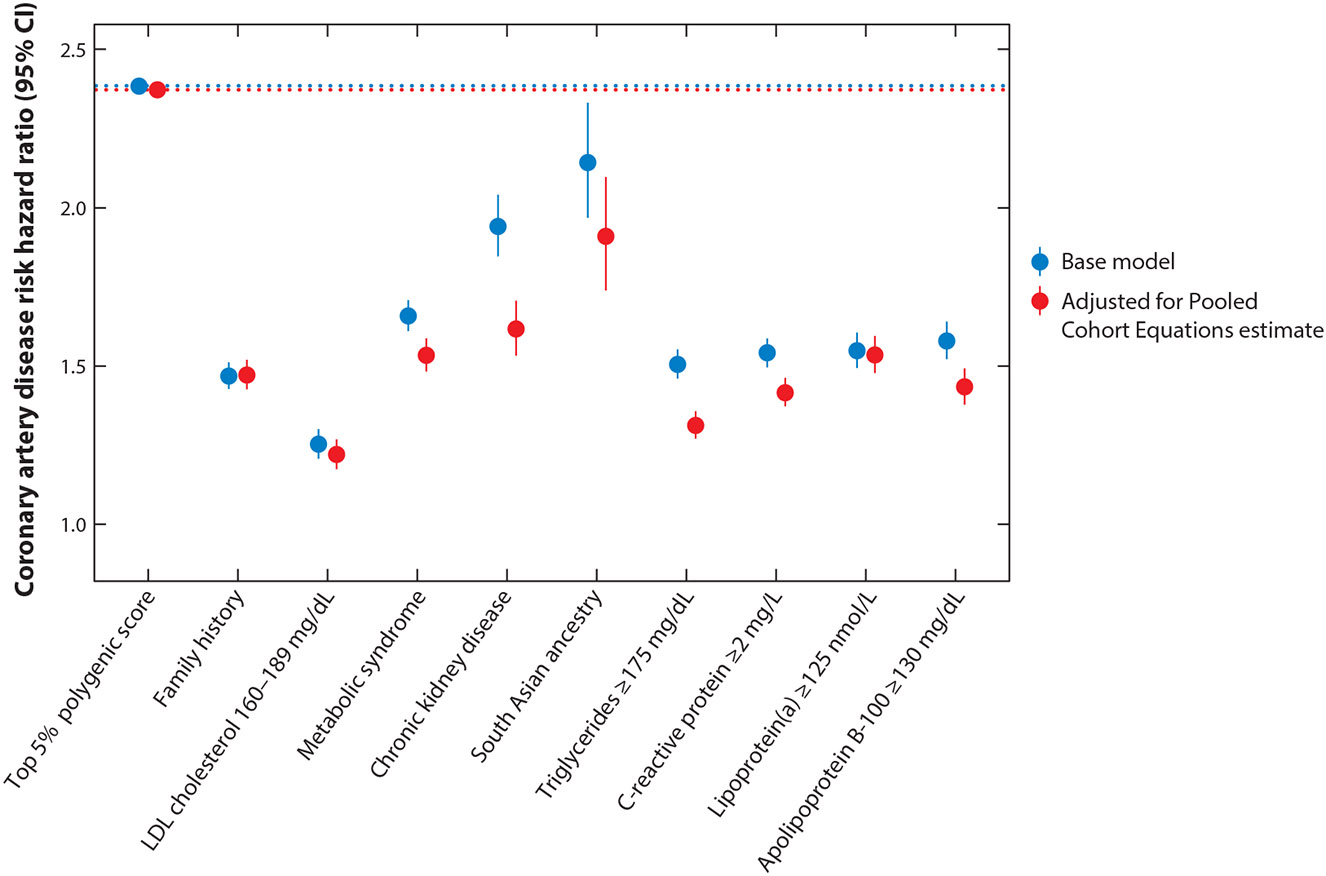
Associations of polygenic scores and risk-enhancing factors with coronary artery disease (CAD). Hazard ratios with corresponding 95% confidence intervals are shown for incident CAD associated with risk-enhancing factors in the UK Biobank, calculated using Cox proportional-hazard models with covariates of enrollment age and sex in base model (*red*), or enrollment age, sex, and Pooled Cohort Equations 10-year risk estimate (*blue*).

Second, moving forward, large-scale randomized controlled trials testing preventative therapies among individuals with elevated genetic risk may help prospectively quantify benefit of use and inform future prevention guidelines. Although most studies of polygenic scores to date have been observational, the evidence collected supporting a high polygenic score as a bona fide risk enhancer is comparable to that supporting other established risk-enhancing factors (Figure 5).

Third, proper interpretation and communication of polygenic scores to clinicians and patients are important additional steps in incorporating polygenic scores into clinical practice. Standards for studies reporting polygenic score results have been established to help facilitate translation into clinical care (54). Several groups have focused on improving the communication and interpretability of polygenic score reports for patients (82, 110). The EMERGE Network study recently launched several efforts to improve the optimal return, understanding, and clinical use of polygenic scores (111). Within the United Kingdom, similar efforts are under way in Our Future Health, which is a nationwide health research program with plans to recruit up to five million participants and with a focus on calculating and communicating polygenic and other risk scores. Direct-to-consumer testing companies have begun offering polygenic score reports for CAD and other diseases to subscribers. With the increase in availability of these data in healthcare system biobanks and patient-ordered testing, future efforts will need to focus on re-education of the clinician workforce (112).

Significant progress has been made from the first observations of inherited mechanisms of CAD to the calculation of polygenic scores to predict disease risk. Their predictive ability has steadily improved with the increase in availability of discovery data sets and more refined statistical genetics methods. Much work remains to be done to improve equity, portability, and integration with available models. Nevertheless, in the era of personalized medicine, polygenic scores are poised to have an important impact on the prediction and prevention of CAD.
